# Supplementary figures and images for: Endothelial cell-specific loss of eNOS differentially affects endothelial function
Source: PLoS One. 2022 Sep 23;17(9):e0274487. doi: 10.1371/journal.pone.0274487 (PMC9506615; doi:10.1371/journal.pone.0274487)

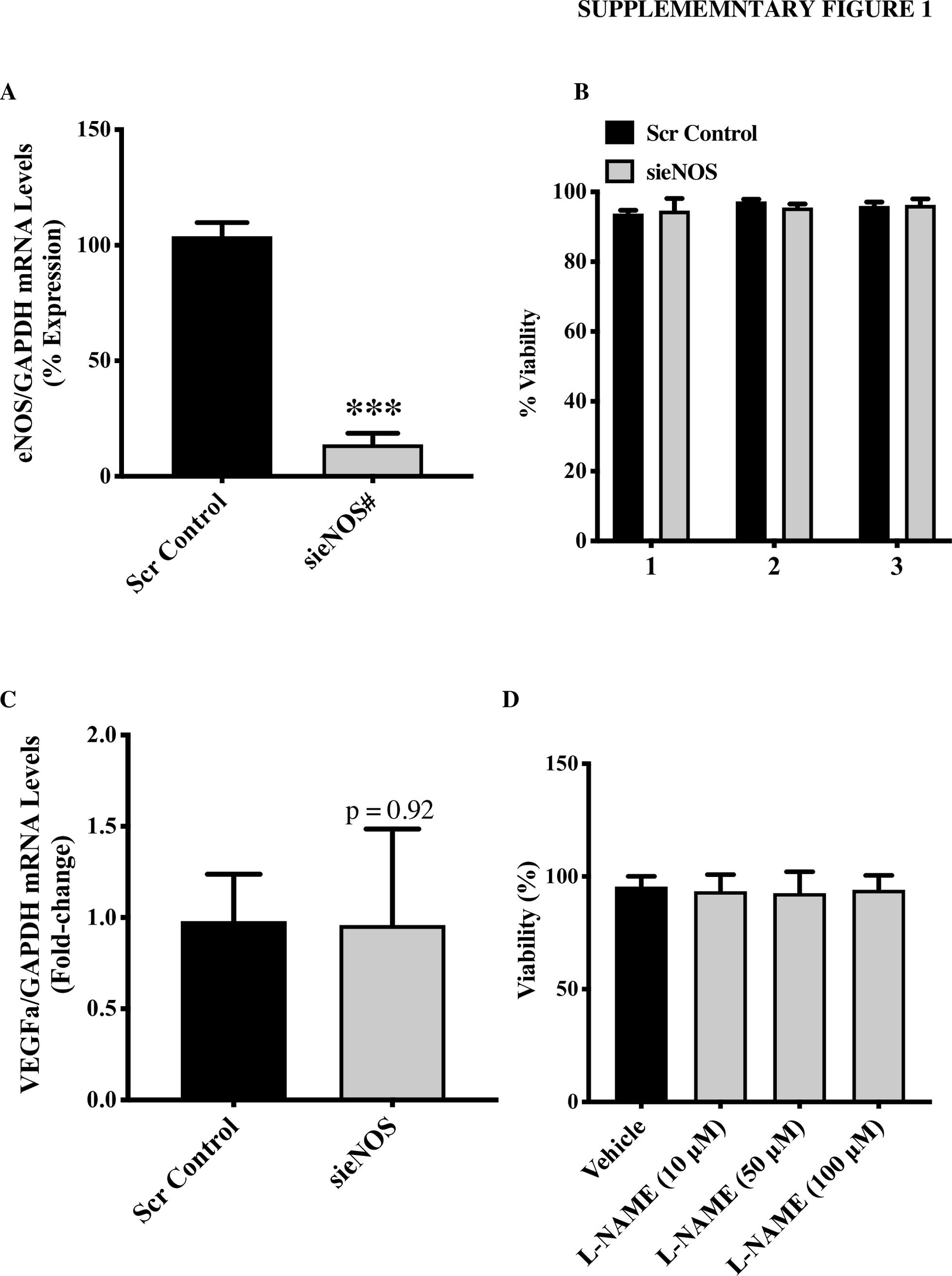

Supplement: S1 Fig — (A) HUVECs were transfected with either scrambled control or sieNOS# (5 nM each) for 24 hrs and RNA was extracted. The knockdown effect was confirmed by qPCR. ***p<0.0001 vs. Scr Control. (B) Cell viability was examined 24, 48 and 72 hrs post-transfection in HUVECs. Each triplicate was counted twice, and average of all triplicates was calculated for each biological replicate. (C) HUVECs were transfected with either scrambled control or sieNOS for 48 hrs and RNA was extracted to perform qPCR for VEGFa. (D) Cell viability was evaluated following 24 hrs of vehicle or different dose of L-NAME treatment in HUVECs. Each triplicate was counted twice, and average of all triplicates was calculated for each biological replicate. (TIF) [file pone.0274487.s001.tif]
