# Supplementary material for: Endothelial cell-specific loss of eNOS differentially affects endothelial function
Source: PLoS One. 2022 Sep 23;17(9):e0274487. doi: 10.1371/journal.pone.0274487 (PMC9506615; doi:10.1371/journal.pone.0274487)
Supplement: S1 Raw images — (PPTX) [file pone.0274487.s002.pptx]

## Slide 1
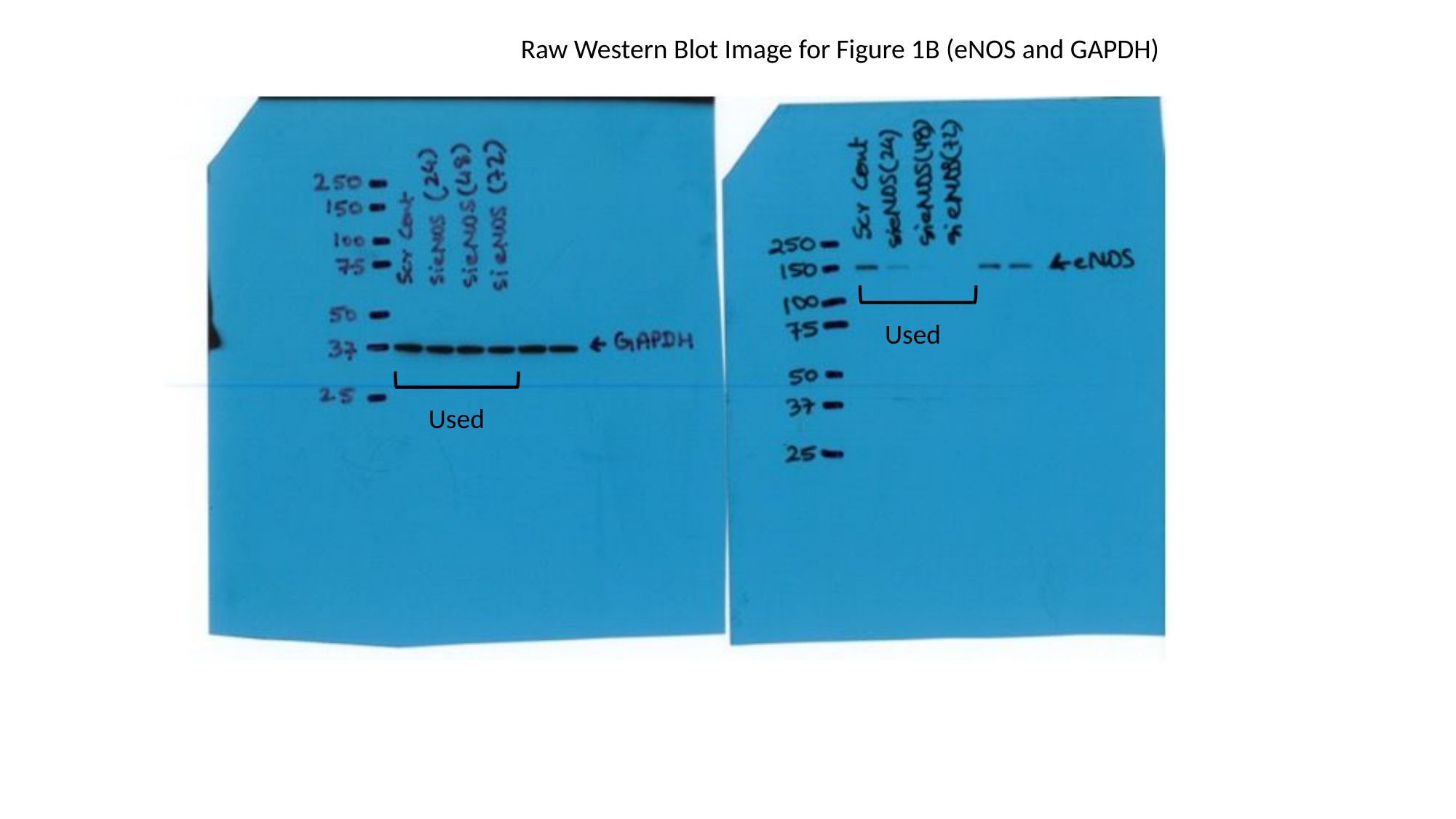

Raw Western Blot Image for Figure 1B (eNOS and GAPDH)
Used
Used

## Slide 2
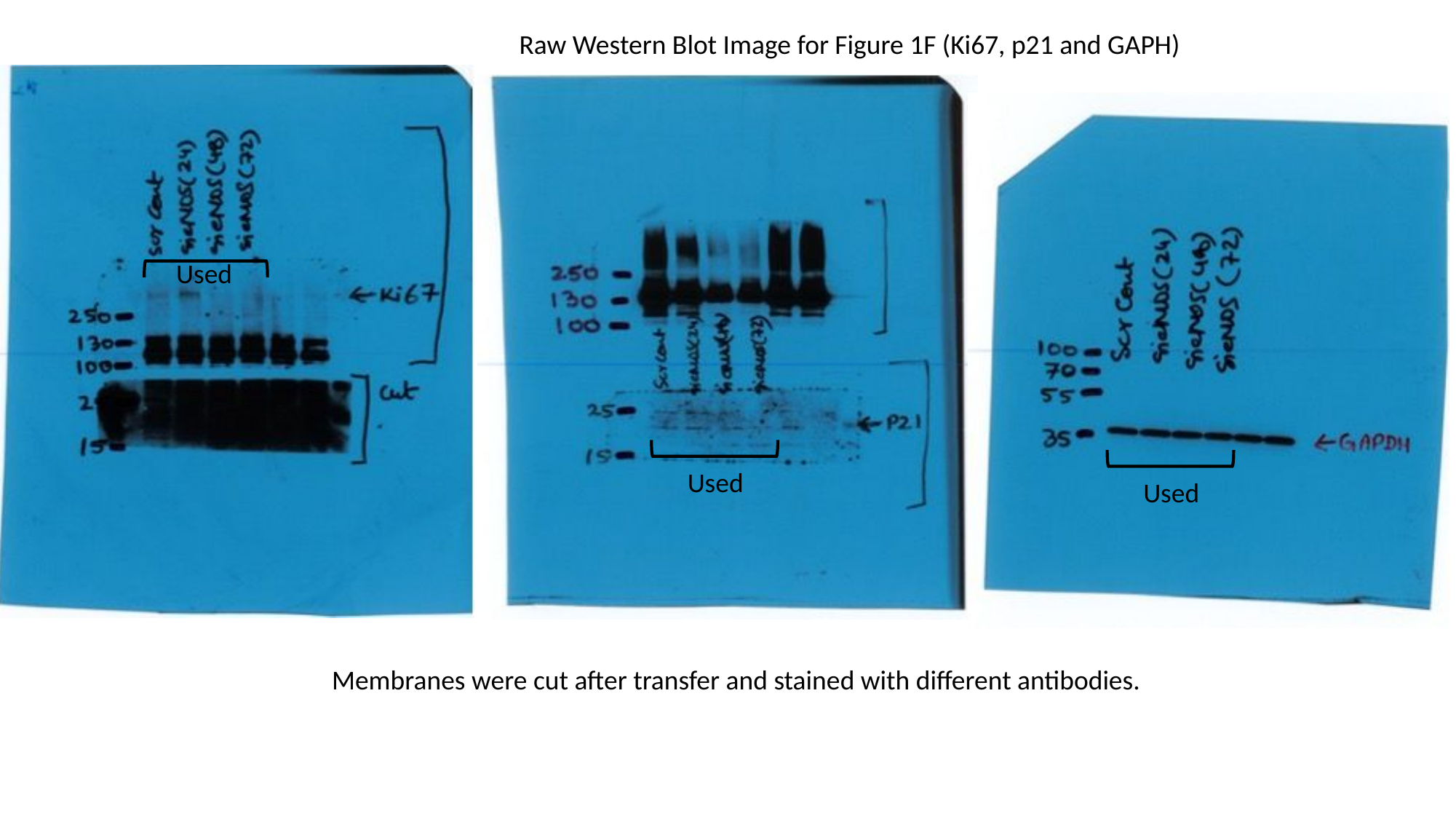

Raw Western Blot Image for Figure 1F (Ki67, p21 and GAPH)
Used
Used
Used
Membranes were cut after transfer and stained with different antibodies.

## Slide 3
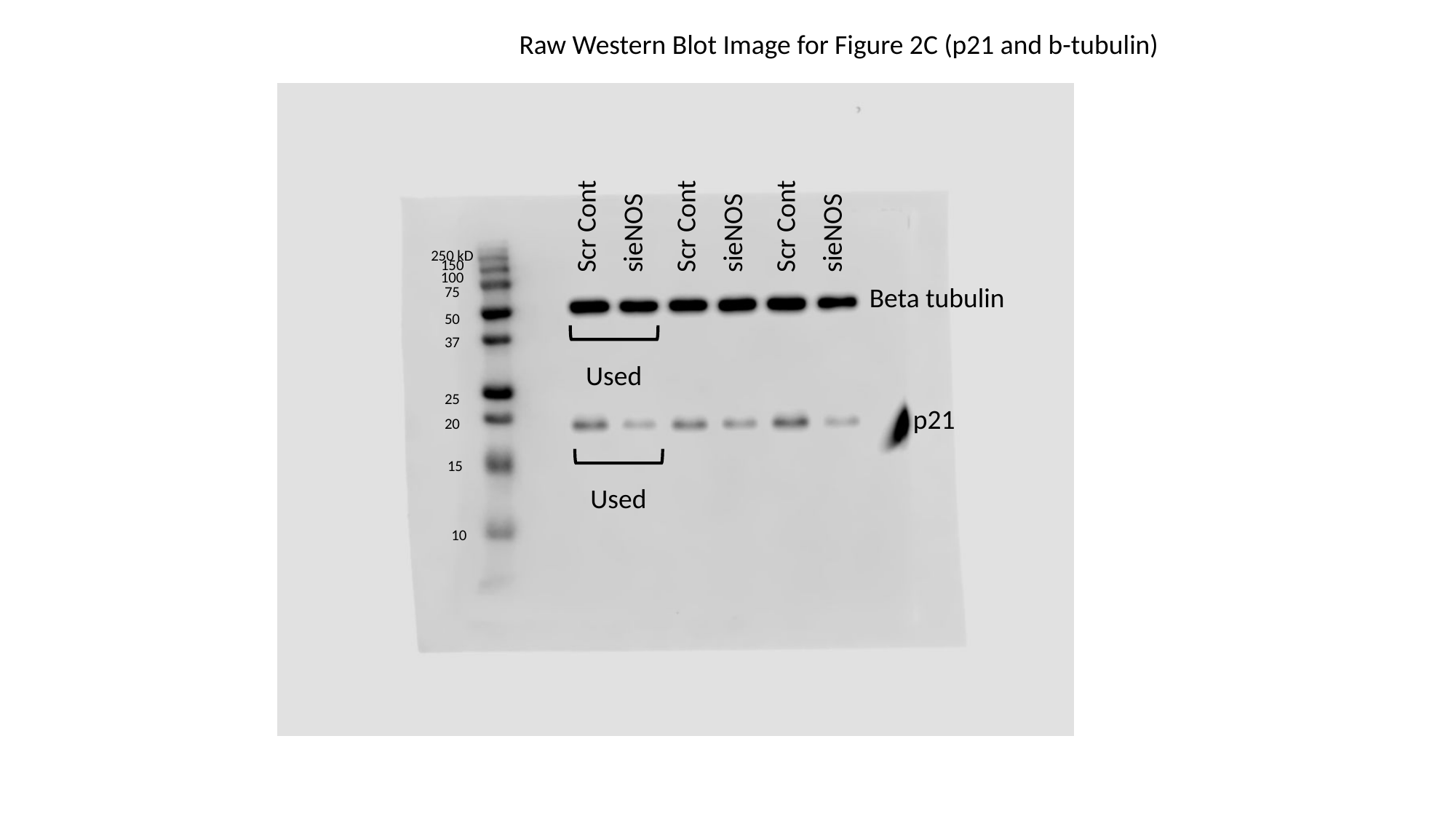

Raw Western Blot Image for Figure 2C (p21 and b-tubulin)
250 kD
150
100
Beta tubulin
75
50
37
25
p21
20
15
10
Scr Cont
Scr Cont
Scr Cont
sieNOS
sieNOS
sieNOS
Used
Used

## Slide 4
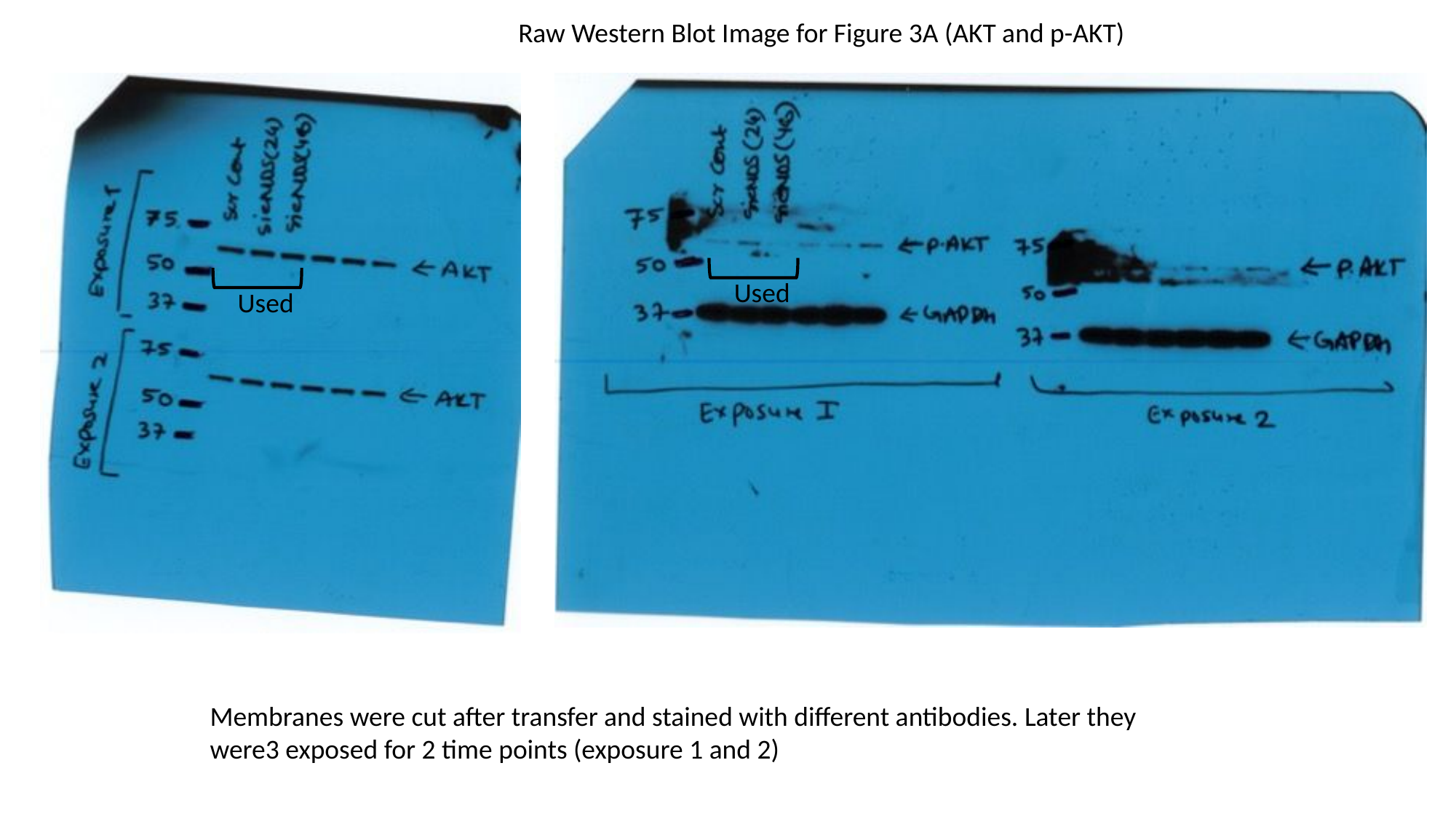

Raw Western Blot Image for Figure 3A (AKT and p-AKT)
Used
Used
Membranes were cut after transfer and stained with different antibodies. Later they were3 exposed for 2 time points (exposure 1 and 2)

## Slide 5
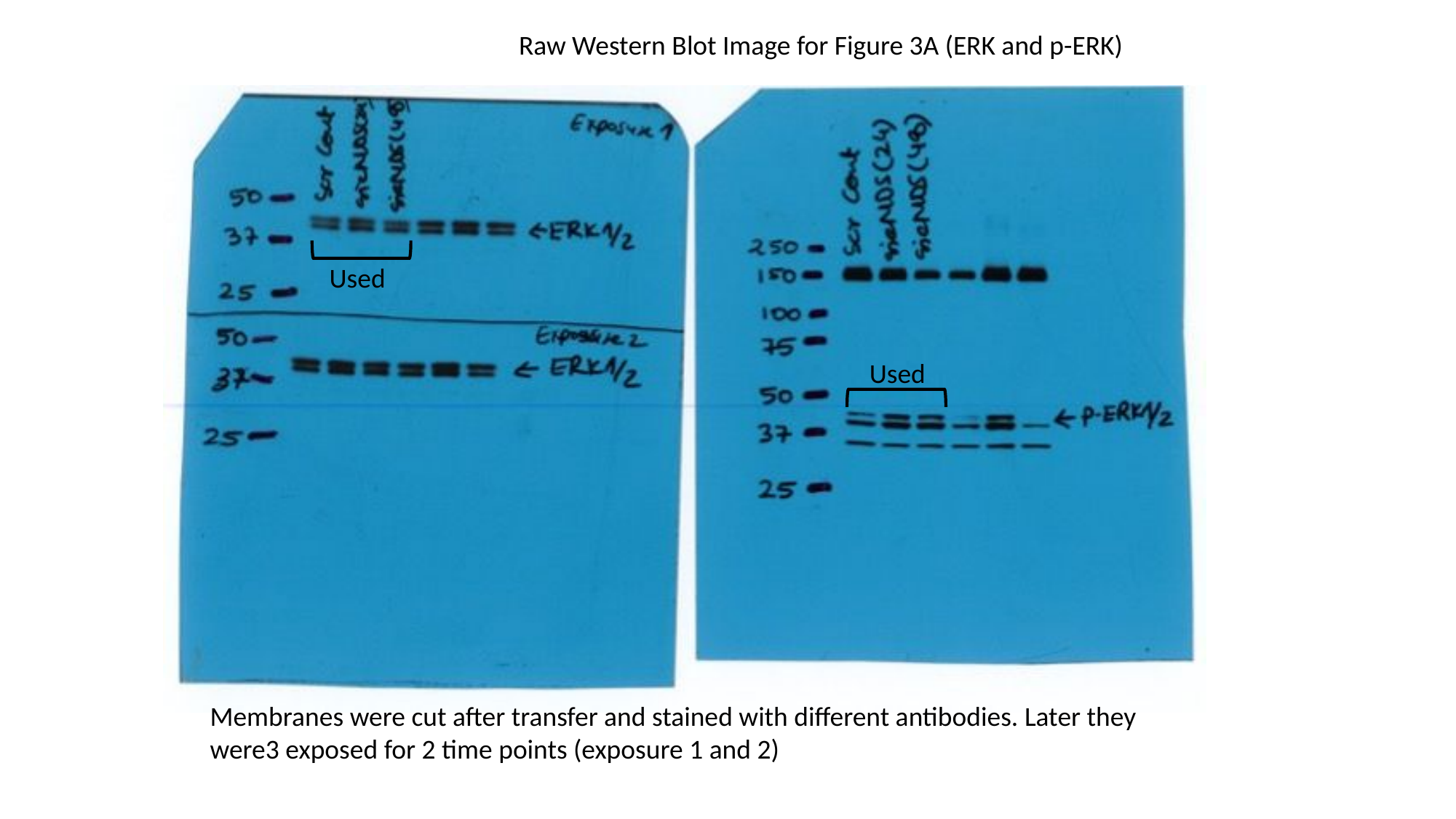

Raw Western Blot Image for Figure 3A (ERK and p-ERK)
Used
Used
Membranes were cut after transfer and stained with different antibodies. Later they were3 exposed for 2 time points (exposure 1 and 2)

## Slide 6
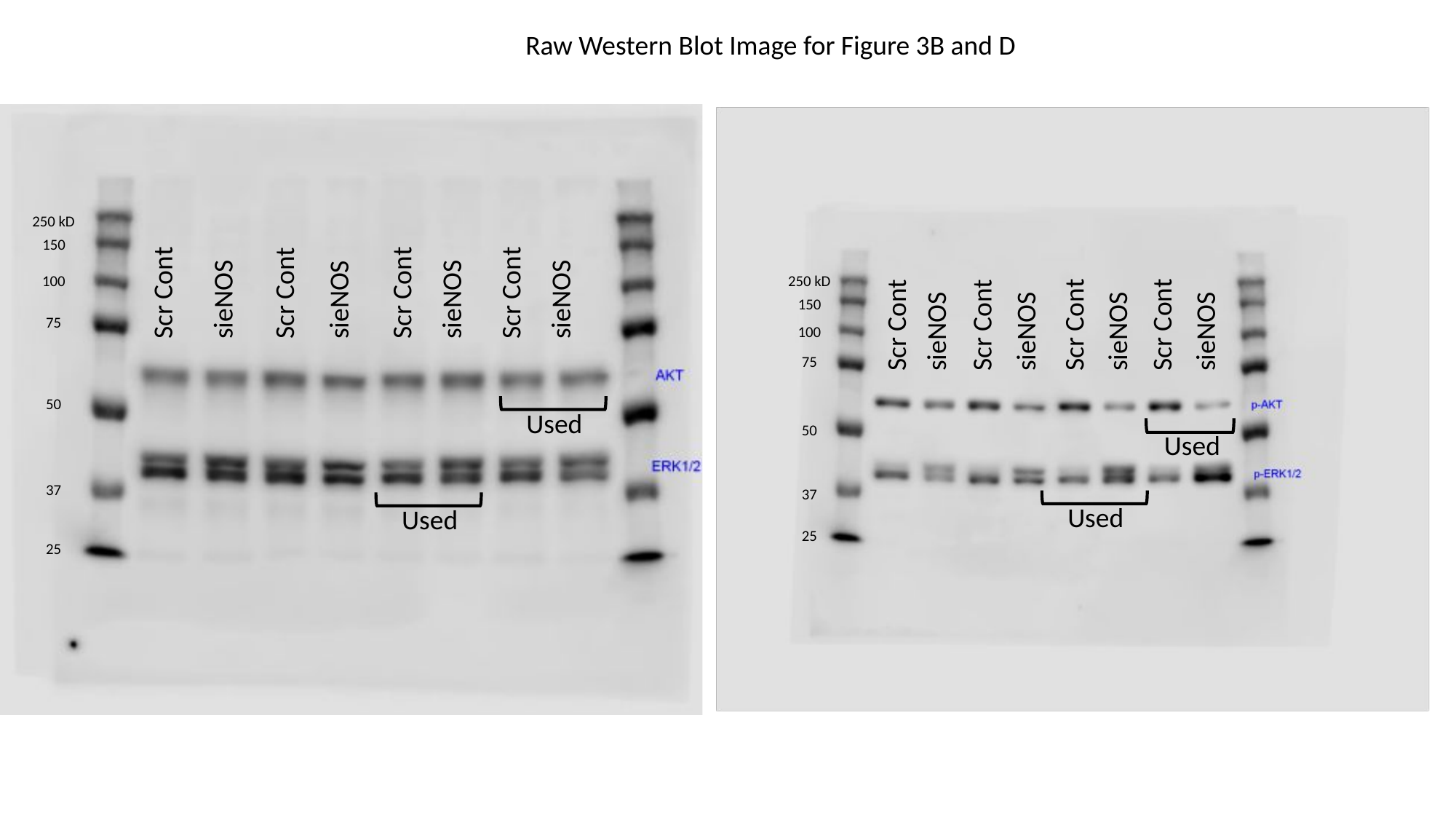

Raw Western Blot Image for Figure 3B and D
250 kD
150
100
250 kD
Scr Cont
Scr Cont
Scr Cont
Scr Cont
sieNOS
sieNOS
sieNOS
sieNOS
150
Scr Cont
Scr Cont
Scr Cont
Scr Cont
75
sieNOS
sieNOS
sieNOS
sieNOS
100
75
50
Used
50
Used
37
37
Used
Used
25
25
