# Supplementary material for: Endothelial cell-specific loss of eNOS differentially affects endothelial function
Source: PLoS One. 2022 Sep 23;17(9):e0274487. doi: 10.1371/journal.pone.0274487 (PMC9506615; doi:10.1371/journal.pone.0274487)
Supplement: Attachment — Submitted filename: Response to reviewers.docx [file pone.0274487.s003.docx]

**Reviewer 1**

**Comment #1 -** Title refer only to proliferation, but Fig 1K also migration.

**Response:** Many thanks for your thoughtful comments. In cultured endothelial cells, loss of eNOS promoted cell proliferation and migration but inhibited tube-forming potential. Accordingly, to have more conclusive title, the revised title of the manuscript is that “Endothelial cell-specific Loss of eNOS Differentially Affects Endothelial Function”.

**Comment #2:** The main question of the paper is quite unclear: Do the authors through proliferation and migration sought to modelize macrovascular endothelial cell healing, or do they sought to modelize angiogenesis? In addition, they refer to eNOS expression in SMC (ref 19) where the mechanisms are totally different, and which pathophysiological meaning is unclear.?

**Response:** We mainly aimed to investigate the effect of loss of eNOS on *in vitro* measures of endothelial function such as proliferation, migration and tube formation, which contribute towards both; endothelial cell healing and angiogenesis. The statement regarding eNOS expression in SMC has been deleted from the revised manuscript.

**Comment #3:** Finally, they report rather preliminary results in nature, and acknowledge that at the end of the introduction “Our findings confirm that eNOS regulate endothelial function by directly controlling endothelial cell proliferation, and non-specificity of L-NAME is responsible for inhibition of endothelial cell proliferation; both warranting further investigation”. Indeed, these results are very descriptive and the amount of limited to 2 Figures. Thus, the apparent « discrepancy » between gene expression inhibition and eNOS activity inhibition should be investigated more in depth.

**Response:** We agree with the reviewer that to some extent the results are descriptive in nature; however, it confirms the effect of loss of eNOS on endothelial function *in vitro*, which has not been clearly reported so far, and that is the focus of the present study. We have plans to investigate the mechanisms behind observed effect, which will be part of our next manuscript. Explanation regarding non-specificity of L-NAME has been deleted from the revised manuscript. We believe that the differential effect of eNOS on endothelial function is observed due to mechanisms up-stream (before eNOS activation) and down-stream (after eNOS activation) to eNOS.

**Comment #5:** PKG increases extracellular concentration of calcium by facilitating the reuptake of cytosolic calcium into the sarcoplasmic reticulum and opening of calcium-activated potassium channels [5]. Ref 5 : Nitric oxide decreases [Ca2+] in vascular smooth muscle by inhibition of the calcium current. Cell Calcium 1994. Is not appropriate as i) it demonstrate in SMC the opposite and ii) the roles of Calcium in SMC and in endothelial cells are very different and cannot be extrapolated.

**Response:** We agree with your thoughtful comment and have deleted the original reference and added new references in Page 3 paragraph 1 of the revised manuscript.

**Comment #6a:** Proliferation and Scratch Assay: the experimental conditions of cell culture should be better defined.

# Response: Thank you for your comment, the experimental conditions have now been further clarified in page 6, paragraph 1 and 2 of the revised manuscript. The migration assay was conducted as described in “An introduction to the wound healing assay using live-cell microscopy” (PMID: 25482647).

**Comment #6b:** L-NAME is in vivo desestherified to L-NA, that is in fact the active coumpound. Thus, Experiments should be done with L-NA and L-NMMA in order to minimize the pharmacological bias and to see if the results if these 3 complementary apporaches do concur. This would allow to attenuate the limitation indicated in the introduction : « non-specificity of eNOS pharmacologic inhibitor L-NAME.

**Response:** Many thanks for your suggestion. Now, we have included data on proliferation and migration following L-NMMA, which is similar to L-NAME data on HUVECS proliferation, migration and tube-formation (Please see Fig. 5 in the revised manuscript).

**Comment #6c:** How many different sieNOS were used? At least 3 should minimize the possible off-target effects.

**Response:** We used two sieNOS (sieNOS and sieNOS#) molecule and then measured proliferation following silencing. However, we observed the similar reduction in proliferation following transfection with both siRNA molecules (Please see supplementary figure 1 and figure 1D).

**Comment #7:** Abstract : “On the contrary,.. “ should be better explained : “at the level of the enzyme activity”.

**Response:** Abstract is revised as suggested.

**Reviewer 2**

**Comment #1:** Authors should use correct term, authors claim that they are 'silencing' eNOS gene. Here, the word 'silencing' is used incorrectly, it should be replaced with 'siRNA mediated knockdown' or simply 'knockdown'. Genetic silencing, by definition, means traditional gene knockout using homologous recombination technology using the embryonic stem cells.

**Response:** Thank you for your comment, it has now been corrected in the manuscript.

**Comment #2**: Can authors provide evidence that eNOS is a negative regulator of cell proliferation? For example, by over-expressing eNOS-cDNA in endothelial cells, which should increase the levels of cell cycle inhibitor such as p21, p27, p53 and down-regulation of Cyclin-D1. These can be done using cultured endothelial cells, at least two different primary endothelial cells must be used. The HUVECs at passage 4-6 are likely not quiescent, therefore, it would be more appropriate to use microvascular endothelial cells such as from lungs and heart. My fear is that the HUVECs may be displaying inappropriate adaptive response, in response to eNOS-knockdown.

**Response:** Thank you for the thoughtful comment. We have followed your suggestion and silenced eNOS in Human Pulmonary Artery Endothelial Cell (HPAECs) and confirmed downregulation of eNOS. Knockdown of eNOS in HPAECS was associated with increased proliferation, downregulation of p21 and enhanced migration as observed in eNOS-deficient HUVECs (Please see Fig. 2 in the revised manuscript). We agree that eNOS-overexpression will very well complement our findings. Accordingly, we have future plans but that will form the basis of another manuscript.

**Comment #3**: Can authors normalize the activity of AKT (pAKT) in endothelial cells that received eNOS siRNA? Thereby rescue/restore the normal EC phenotype? Alternatively, can authors add back eNOS-cDNA to restore normal phenotype?

**Response:** As suggested, we have normalized the AKT activity. Please see figure 3B and C.

**Comment #4**. Figure 1L: Quantification of Western Blot is required.

**Response:** As suggested, we have quantified AKT and ERK protein levels. Please see figure 3B-E.

**Comment #5**: The migration assay (wound healing) done over a period of 20 hours could include cell proliferation as well. Did author add BrdU during cell migration assay? This data is unclear.

**Response:** Thanks for your comment. We incubated cells in DMEM supplemented with 1%FBS to ensure there is only migration but not proliferation. Method is now clarified in page 6 paragraph 2.

**Comment #6**: In the Figure legend, can authors mention as to how many times experiments were repeated? Please explain N value clearly.

**Response:** Thank you for the comment. We have repeated the experiments at least 3 times in triplicates and the N values are now added in the figure legend.

**Comment #7**: 'Tube formation' assay is misleading. Can authors indicate where the tubes are?

**Response:** Thanks for pointing it out. As suggested, tubes are marked in the figure 1G of the revised manuscript.

**Comment #8**: Methods are described inadequately.

**Response:** Thank you for the comment, we have added more details to the methods section.

**Comment #9:** 'matrigel' should be spelled Matrigel, where 'M' should be capitalized.

**Response:** Thanks for the reminder, it has now been corrected.

**Comment #10:** The manuscript could benefit from check on English grammar and clarity.

**Response:** Thanks for the suggestion. We have now checked the manuscript for grammar and clarity.
