# Supplementary material for: Endothelial cell-specific loss of eNOS differentially affects endothelial function
Source: PLoS One. 2022 Sep 23;17(9):e0274487. doi: 10.1371/journal.pone.0274487 (PMC9506615; doi:10.1371/journal.pone.0274487)
Supplement: Attachment — Submitted filename: Response to Reviewers August 25.docx [file pone.0274487.s004.docx]

**Reviewer #1:** Seems fine no additional comment Seems fine no additional comment Seems fine no additional comment Seems fine no additional comment

**Response:** Thank you very much.

**Reviewer #2:** The authors have addressed most of my questions and concerns.

However, the use of terminology remains confusing, e.g., tube formation. If authors claim that these are indeed tubes (Figure 4B, indicated by arrows), the Matrigel must be fixed with paraformaldehyde (4% PFA) and embedded in paraffin, make cross thin section, thereafter stain with H&E and show lumen (vacuole/empty space). In other words, "lumen formation" is alternatively called "tube formation".

**Response:** Thank you for your insightful comment. We agree that endothelial cells link to each other through formation of junctional complexes upon stimulation of angiogenic signals which lead to formation of lumen, and fixation will help visualize the lumen. We have now changed the “tube formation” to “formation of tube-like structures”.
